# Difference between Doctors and Lawyers

**Published:** 1854-11

**Authors:** 


### DIFFERENCE BETWEEN DOCTORS AND LAWYERS.

Judge Daly, at a late meeting of the Society for Relief of Widows of Physicians, said:

I doubt if as good an understanding subsists among members of the medical as among those of the legal profession, and this, I think, may be attributed to the different manner in which they pursue their different vocations. The lawyer is always pitted against an adversary; he is brought at once into collision with a skillful and well-armed opponent, in a mutual struggle for victory. For the display of his powers and the exercise of his talents, he comes into a public arena and champions his adversary to the combat. Now the effect of this collision is highly beneficial and healthful. As nothing is more dangerous than pent-up passion or long-nursed animosities, an opportunity is here found to give them vent; they consequently evaporate in this war of words, and, whatever sharp things lawyers publicly say of each other, the matter ends with a healthier feeling and a better understanding. "We always have a better feeling for a man," says an old Greek philosopher, "after we have wrestled with him," and such is the effect of this legal wrestle. It is a kind of purging process, stirring up all the bile and ill humors of the system, and throwing them off by means of this healthy agitation. There is, moreover, a great advantage in having somebody to act as an umpire between them to decide the matter, and put an end to the strife. But, says the world-wide adage, "who shall decide when doctors disagree?" Consequently, Mr. Chairman, it is difficult to find a more social set, when they come together, than a nest of lawyers. Now, the case of the physician is very different. He is a silent and a solitary worker. The matter is entirely between himself and his patient. He is, consequently, more exposed to the carping of professional envy and to the misrepresentation of professional jealousy. He is more easily assailed, and less easily defended. He is powerless himself, for he cannot champion his undervaluer to the lists, and overthrow him in the contest of professional skill. He has no shrewd and keen adversary to take advantage of the faults, or, what may be of more value to him, to appreciate and feel his ability. There are no echoing plaudits to hail the triumphs of his genius. For the development, therefore, of those social feelings that grow from professional intercourse, his position is far less advantageous than that of his legal brother.

